# Active backlight for automating visual monitoring: An analysis of a lighting control technique for *Caenorhabditis elegans* cultured on standard Petri plates

**DOI:** 10.1371/journal.pone.0215548

**Published:** 2019-04-16

**Authors:** Joan Carles Puchalt, Antonio-José Sánchez-Salmerón, Patricia Martorell Guerola, Salvador Genovés Martínez

**Affiliations:** 1 Universitat Politècnica de València, Instituto de Automática e Informática Industrial, Valencia, Spain; 2 Cell Biology Laboratory/ADM Nutrition/Biopolis SL/Archer Daniels Midland, Paterna, Valencia, Spain; Imperial College London, UNITED KINGDOM

## Abstract

Lifespan and healthspan machines can undergo *C. elegans* image segmentation errors due to changes in lighting conditions, which produce non-uniform images. Most *C. elegans* monitoring machines use backlight techniques based on the transparency of both the container and media. Backlight illumination obtains high-contrast images with dark *C. elegans* and a bright background. However, changes in illumination or media transparency conditions can produce non-uniform images, which are currently alleviated by image processing techniques. Besides, these machines should avoid *C. elegans* exposure to light as much as possible because light stresses worms, and can even affect their lifespan, mainly when using (1) long exposure times, (2) high intensities or (3) wavelengths that come close to ultraviolet. However, if short exposure of worms to light is required for visual monitoring, then light can also be used as a movement stimulus. In this paper, an active backlight method is analysed. The proposed method consists of controlling the light intensities and wavelengths of an illumination dots matrix with PID regulators. These regulators adapt illumination to some changing conditions. The experimental results shows that this method simplifies the image segmentation problem because it is able to automatically compensate not only changes in media transparency throughout assay days, but also changes in ambient conditions, such as smooth condensation on the lid and light derivatives of the illumination source during its lifetime. In addition, the strategic application of wavelengths could be adapted for the requirements of each assay. For instance, a specific control strategy has been proposed to minimise stress to worms and trying to stimulate *C. elegans* movement in lifespan assays.

## Introduction

The tiny nematode worm *Caenorhabditis elegans* (*C. elegans*) offers us a window into biology because they allow researchers to track in vivo biological events [1, 2]. The ease with which *C. elegans* can be grown, manipulated and observed has driven research to new areas.

Different in vivo assays can be run in which *C. elegans* models are used to analyse their phenotypes, through which toxicity factors can be inferred to certain compounds, such as therapeutic factors to some neurodegenerative diseases, alterations in ageing, etc. As part of this wide variety of assays, two models interest us, Lifespan and Healhspan, for which the motility phenotype is the most widely observed. The Lifespan model [3–10] measures the survival percentage of samples submitted to different conditions to infer which factors alter life expectancy. Survival is determined by worm movement existence (life) or its lack of movement after poking (death). In the particular case of the lifespan assay with *C. elegans*, it is known that maximum life is about 3 weeks, and lasts a few weeks more for some strains, which make experiments shorter than for other animals. Healthspan [11–13] studies the nematode life quality by observing animal motility features. Reduced movement or its lack of coordination can be due to causes such as ageing, neurodegenerative diseases, intoxication, muscle problems, etc. These assays need about 100 specimens per condition, which entails a huge workload for researchers to handle them, count them and measure their features. In addition, some of these tasks have to be done daily, which means arduous work. This is why a need arises to automate these assays. Apart from saving researcher time, automated technology also promises objectivity, constant monitoring and new measures of worm features.

There are a number of researcher groups worldwide that are developing new technologies to automate *C. elegans* monitoring tasks. Each technology uses a different methodology, but the common subject is to detect worm movement by imaging at the mesoscale level when monitoring standard Petri plates completely [14–17].

Full automation of lifespan and healthspan experiments is a challenging problem because the images captured during assays can present spatial and temporal variability. Spatial variability in an image can be due to different *Escherichia coli* or tested compound concentrations, contaminated areas, lint from the outside on caps (which can adopt quite similar shapes to worms), light refractions on Petri walls, etc. Despite worms’ short lifespans, temporal variability remains for various reasons: biological changes (worms feeding, worms defecation, worm tracks, *E. coli* growth, contamination, etc.), changes in temperature (agar evaporation, internal condensations on lids, etc.) and derivatives from devices (illumination derivatives, differences in Petri plate location on different days, sensor sensitivity changes, etc.). The present study pays attention to the *C. elegans* segmentation problem on standard Petri plates, which are due mainly to: (1) medium changes; (2) internal condensations on lids; (3) illumination source derivatives. These variations in lighting conditions could affect the image quality in some areas, and could imply losing some information that might not be recovered with conventional segmentation algorithms.

In the last few years, several software techniques have been used to correct these uneven lighting errors in assays run with *C. elegans* for capturing images and subsequently processing them. Usually a threshold is tuned manually during the assay. However, automatic adaptive methods are used by, for example, [18–20], and adaptive Gaussian is also employed [21], as is Otsu’s method [22, 23], which is a clustering-based image and a method based on contrast [24]. There are also graph methods [25, 26] or background modelling [27]. Another technique is edge [28, 29], which detects image gradient magnitude. Filling object areas is usual for improving the segmented image by using dilate and erode methods [30, 31] or filling operations [31].

From our point of view, instead of solving this problem manually or via software, it is better to solve it by hardware with an intelligent illumination system that is able to adapt illumination to changing conditions. This solution must be robust for some changing conditions by simplifying the image segmentation problem. Nevertheless, as far as we know, none of the state-of-the-art monitoring systems has actively controlled its light.

Besides, it has been demonstrated that all visible light wavelengths are lethal for transparent *C. elegans*, especially when applying (1) long exposure times, (2) high intensities and/or (3) wavelengths that come close to ultraviolet [32]. However, this mortality becomes insignificant if light intensity is reduced to units less than *μW*/*mm*^2^ and the exposure time is cut to a few seconds per day. Exposure of worms to light leads to withdrawal responses [33, 34], mainly if blue light is applied. WorMotel [35] used a non-controlled red light to detect movement and emitted a blue flash as a stimulus to force movement behaviour in lifespan assays, instead of using illumination with on/off control, which affects the whole plate. In our view, it could be very interesting to control light intensities, exposure times and wavelengths in small zones of the plate as and when desired.

In this paper, an active backlight method is proposed for lifespan and healthspan machines that work with transparent Petri plates and media as it allows precise automatic illumination control. We analyse a control technique based on active backlight capable of regulating dot-to-dot intensity light and its wavelength (Red, Green, Blue). The proposed method consists of controlling the light intensities and wavelengths of a matrix of illumination dots with PID regulators. These regulators obtain feedback from captured images to adapt illumination to changing conditions. The control references are the desired image intensities. These references are set to the lowest intensity values to allow robust segmentation for minimising worms stress. In addition, a new control action strategy is proposed to take advantage of the wavelength control to stimulate *C. elegans* in an attempt to improve lifespan results.

## Materials and methods

### Materials

**Fig 1:**
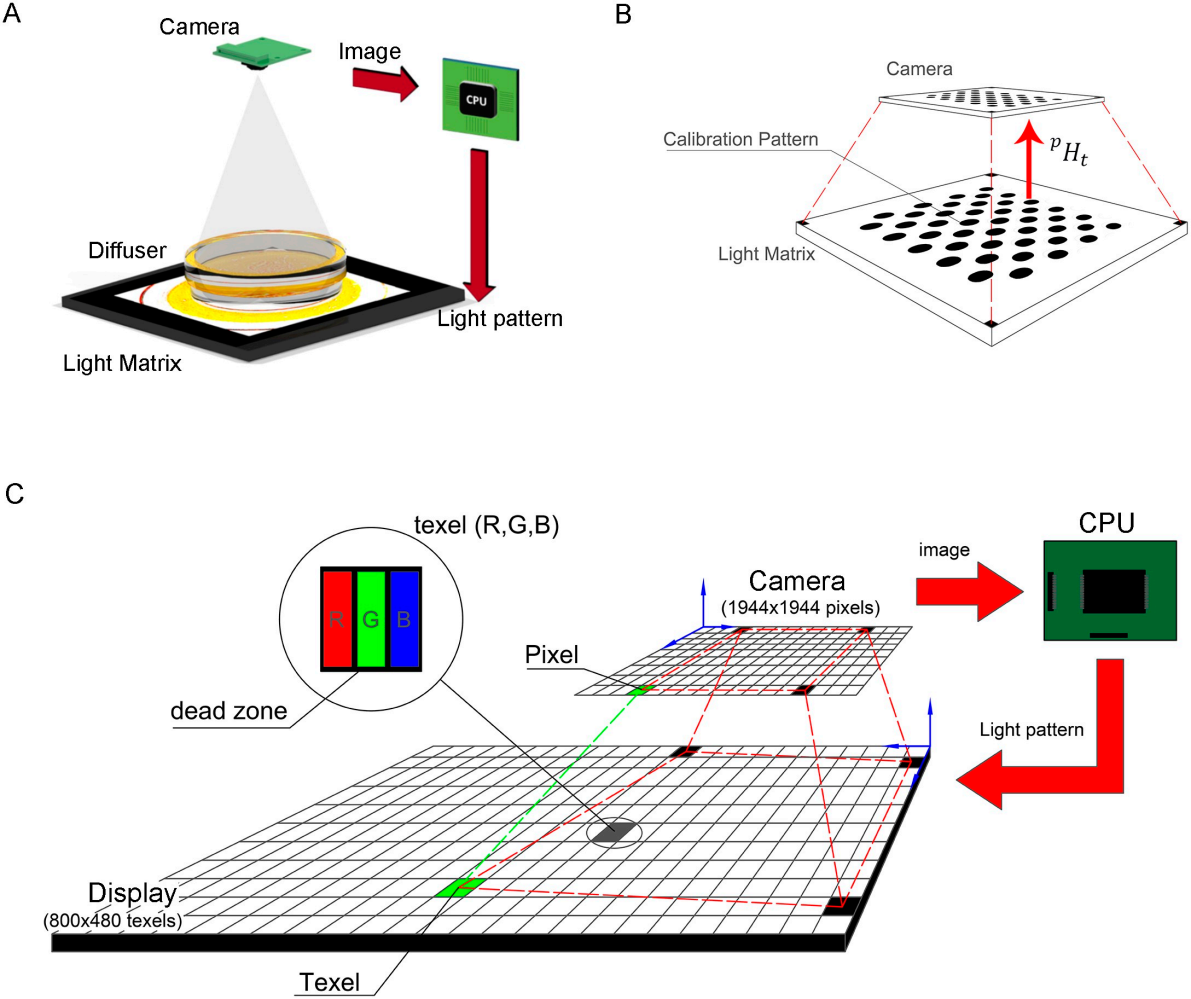
Physical configuration. A: Backlight scheme. B: Homography calibration scenario. C: Physical features of some components.

Different illumination techniques can be applied to monitor behaviours of the worms cultured on standard Petri plates. These techniques are defined by the location in relation to the illumination device, the inspected plate and the camera. A backlight configuration consists of placing a camera in front of the illumination system and the inspected plate in between. In our case, the inspected subjects are *C. elegans* cultured on a 55-mm Petri dish with NGM (Fig 1A).

A matrix of light dots is required to develop a spatial control, with which intensity and wavelength can be controlled dot-by-dot. Each dot is composed of some subdots of different wavelengths. In our case, these subdots are red, green and blue (R, G, B). There are dead zones between these dots where no light could be emitted, which produces a dark reticular structure on captured images. To avoid this problem, we propose using a diffuser, which is placed on top of the light matrix. On the diffuser, we place the Petri dish in such a way that the light emitted by the dots crosses the transparent Petri dish and media towards the camera that captures high-contrast images with dark *C. elegans* and a bright background. The camera, besides being used to capture images for monitoring purposes, also serves as the control loop feedback sensor to allow the lighting of each dot to be regulated.

Moreover, in order to control lighting, it is necessary to isolate the system from the environment to reduce ambient light disturbances. Consequently, the system is enclosed in a box, which is not depicted in Fig 1A. In addition, the interior of this box is covered with black material to avoid internal reflections.

We have developed a low-cost system as a proof-of-concept. However, this system can be made using other materials and components. Our system is composed of the 7” Raspberry Pi Display with 800 × 480 texels as the light matrix, a glass diffuser, 55-mm Petri dishes, a Raspberry V1.0 camera (Pi camera) and a Raspberry Pi 3 processor.

### Lighting and camera configuration

The first step for system configuration (Fig 1A) is to obtain the camera working distance to the dish (77.5mm) in order to observe the whole dish in an image of 1944 × 1944 pixels, which is determining from the smallest camera angle of view (21°) and then by focusing the lens for that distance to make the image as sharp as possible.

The display can emit three different wavelengths (R, G, B) from each dot or texel (Fig 1C). However, the camera does not distinguish among these wavelengths because it is configured as a grey camera. Therefore from the camera’s point of view, the three wavelength intensities are integrated into a pixel as grey intensity. We seek an operation point in which lighting should be as dim as possible to minimise stress for worms.

In order to avoid a chaotic system in control, the automatic camera settings are disabled. We tune two configuration parameters (integration time and brightness). The integration time must be quite high to improve image quality in a low illuminated scenario. Brightness is irrelevant for image quality because it also affects noise. To obtain optimal parameters, we place a Petri dish with *C. elegans* and look for integration time values, which make the maximum contrast and sharpness between worms and the background for the given illumination. When finding the best integration time for that light, the same search is performed for different illumination intensities.

After numerous tests, we verified that the optimum operating point was given by an illumination that came close to orange (R = 255, G = 190, B = 0), an integration time of 100 ms and a brightness of 25, which gave the background image with an intensity level of 48 and the worm intensity near the 0 level.

### Calibration

Camera calibration is a mature procedure [36]. Several camera calibration techniques exist, but the present paper focuses on the calibration method based on a bi-dimensional pattern. In this work, the open source software library OpenCV is used. It offers three types of calibration patterns: symmetric, asymmetric and checkboard. Some studies, such as [37], have established that patterns of circles are less sensitive to blurring than a calibration checkerboard. We use an asymmetric pattern of circles (Fig 1B) to calibrate the projection matrix *^p^H_t_* (Eq (1)). In our case, this projection is a homography, a 3x3 matrix. This transformation defines the mapping between texels (points defined in relation to the coordinate system of the illumination system) and pixels (points defined in relation to the camera’s coordinate system).

$$\begin{array}{c} y_{k}={}^{p}H_{t}\cdot texel \end{array}$$ (1)

The OpenCV calibration tool runs an automatic circle recognition procedure. Circles recognition is based on the well-known OpenCV BLOB (binary large object) detection method. This consists of calculating the connected blob centroids with sub-pixel precision. The blob detection method also allows the filtration of returned blobs by colour, area, circularity, etc. The default values of these filter parameters are tuned to extract dark circular blobs. In general, OpenCV calibration can be run without having to adjust these default parameters, but the default values in our specific research had to be adjusted to detect circles.

### Controller

Research works to regulate the amount of illumination at a constant level have been previously performed [38, 39]. These works demonstrate that a simple model for each zone can be used to control lighting by a PID regulator. In our case, the dynamics and non-linearities were negligible.

The control references (*ref*) were established to an intensity level of 48 because it is the calculated optimum operation set point. Therefore, the controller was designed around a nominal light amount of 58% (255, 190, 0).

The output (*y*_*k*_) was measured by the intensity value of the image pixel (Eq (2)), where *k* is the index of each sampling time (*T*_*sa*_ = 0.11*s*). To avoid the transformation product matrix calculation for each texel in every iteration, a lookup table was used.

$$\begin{array}{c} y_{k}=Image_{k}\left({}^{p}H_{t}\cdot texel\right) \end{array}$$ (2)

**Fig 2:**
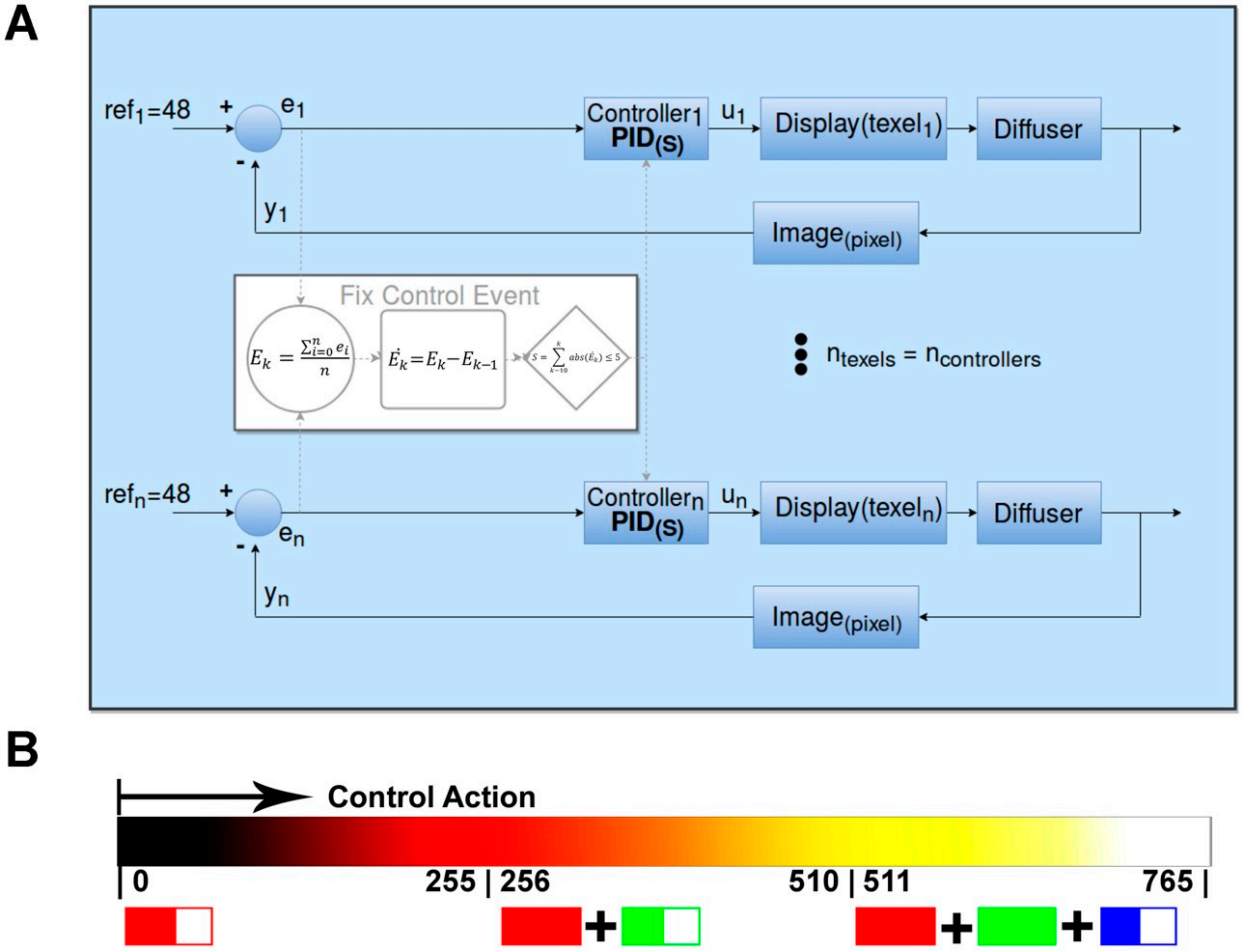
Control principle. A: Control scheme. B: Control action going from black, red, orange, yellow to white, from the minimum level (zero) to the maximum level (255R+255G+255B = 765).

The controller was estabilised when the null control error was reached (*e*_*k*_ = 0), which meant that the intensity output reached the reference level (*y*_*k*_ = *ref*). In order to achieve this goal, a PID control action (*u*_*k*_) was implemented for each texel (Fig 2A). A PID controller (Eqs (3) and (4)) had three parameters: *k*_*p*_ (proportional constant), *k*_*i*_ (integral constant) and *k*_*d*_ (derivative constant). In our case, the *k*_*d*_ constant was set at 0 to obtain a proportional and integral regulator. Our application required a moderate settling time (*T*_*se*_ < 15*s*). Therefore to simplify our controller, we proposed *k*_*i*_ = *k*_*p*_ (Eq (5)) by obtaining a regulator of one degree of freedom. In our case, proportional constant *k*_*p*_ was tuned experimentally to become the maximum positive value, which gave a stable output response (*k*_*p*_ = 0.9).

At each sampling time *k* and for each texel, one controller reads the corresponding output intensity level (*y*_*k*_) from the captured image and applies the control action (*u*_*k*_), which depended on the previous control errors, to close the loop.

$$\begin{array}{c} e_{k}=\left(ref-y_{k}\right) \end{array}$$ (3)

$$\begin{array}{c} u_{k}=k_{p}\cdot e_{k}+k_{i}\cdot \sum_{j=0}^{j=k-1}e_{j}+k_{d}\cdot \left(e_{k}-e_{k-1}\right) \end{array}$$ (4)

$$\begin{array}{c} \text{let}\;k_{i}=k_{p}\;\text{then}\;u_{k}=k_{p}\cdot e_{k}+u_{k-1} \end{array}$$ (5)

A control action (*u*_*k*_) was proposed as the integration of the three wavelength intensities after taking into account a strategic order. This control strategy started when using only red intensity until saturation took place at the 255 level. It continued by adding green intensity and finally blue intensity when green was saturated (Fig 2B). The control action increased progressively from black, red, orange, yellow to white. It should be noted that control strategy used blue light as the last option to reach the reference. If this control strategy was not interesting for a specific assay, it could be easily changed to apply only red light, white light, or others.

### Fixing the control action strategy

There are a number of control loop iterations after which a stable output was achieved. In our case, the control loop was run until the control error was estabilised at a low value. The control action was fixed when a low control error was detected (fixed control event). This event was defined by *S* ≤ 5 (Eq (8)), where *E*_*k*_ (Eq (6)), is the average total error per image at each sampling time *k*; *n* is the texels number of the lighting pattern; $\dot{E_{k}}$ (Eq (7)) is the differential of *E*_*k*_. This event was detected when the integral of the last 10 instants of the $\dot{E_{k}}$ came close to zero (*S* ≤ 5).

$$\begin{array}{c} E_{k}=\frac{\sum_{i=0}^{n}e_{i}}{n} \end{array}$$ (6)

$$\begin{array}{c} \dot{E_{k}}=E_{k}-E_{k-1} \end{array}$$ (7)

$$\begin{array}{c} S=\sum_{k-10}^{k}abs(\dot{E_{k}})\leq 5 \end{array}$$ (8)

## Experiments and results

**Fig 3:**
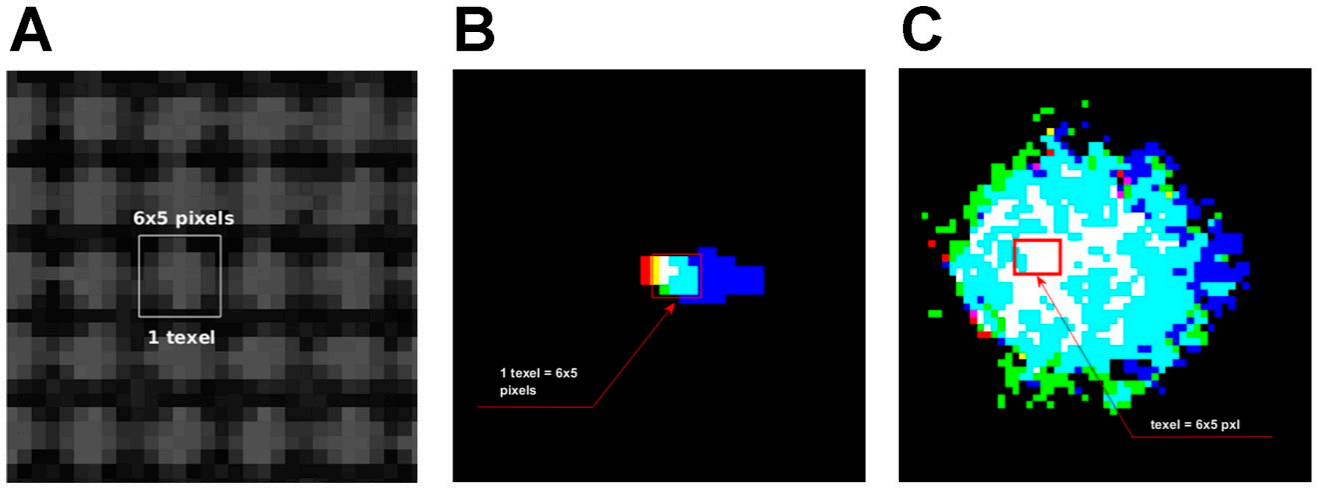
Textel projection on image. A: Greyscale image of white illuminated texels with no diffuser. The white box is the image of one texel acquired by the camera. Both images (B and C) show non-zero wavelength intensity values instead of intensities values. B: Image of the each RGB channel of one texel with no diffuser. C: Image of one texel with a diffuser.

The homography mapped each texel with the central pixel of its projected area on the image. The reprojection error (RE) was used to measure the calibration quality assessment. RE was defined as the geometric error corresponding to the average image distance, measured in pixels, between a texel point, and its projection according to the calibration model, and its corresponding measured counterpart. The retroprojection errors obtained at different calibrations fell within the 2.50 ± 0.06 pixels interval. According to these calibrated homographies, a texel projected approximately on an area of 6 × 5 pixels on the image when no diffuser was used, as seen in Fig 3A. The intensity on the image caused by texel was maximum in the projected area centre, which is the integration of the three emitted RGB wavelengths (Fig 3B). There were control dead zones between texels, which is why a dark reticular structure is observed in the image (Fig 3A).

**Fig 4:**
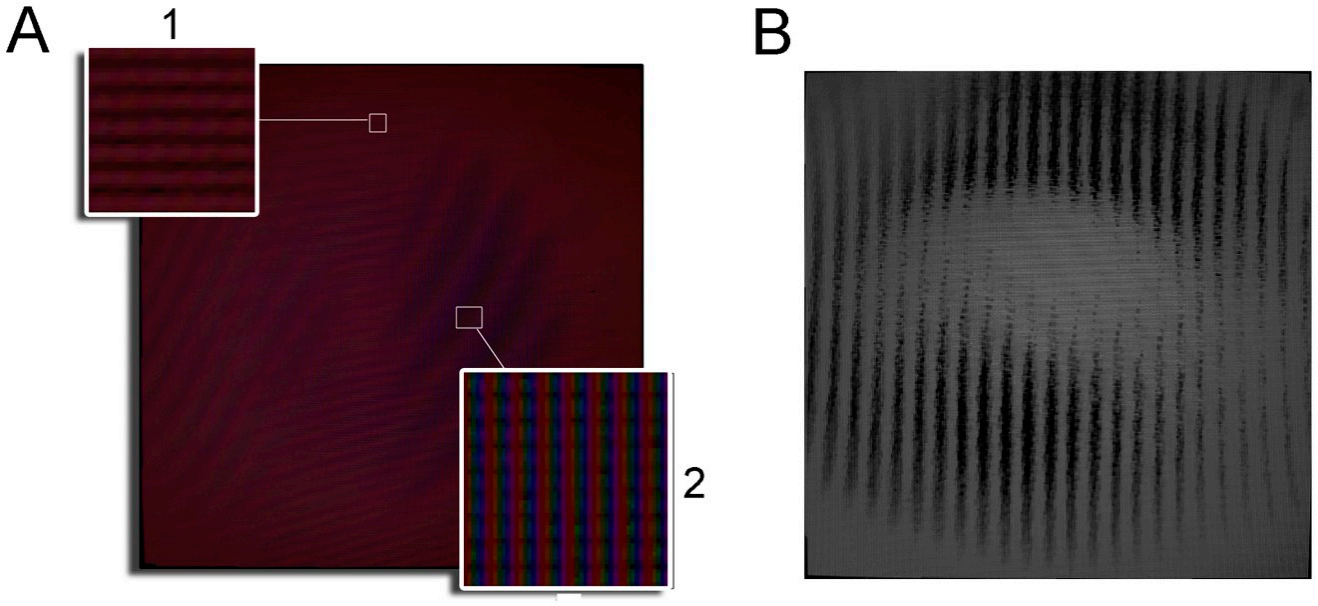
Texel colour dispersion. A: Three images were taken with no diffuser, one with each displayed texel illuminated in red, another with all the texels displayed in green and another one, all blue. All these captures was merged in this image at three layers, RGB. Thus we can see for each image pixel how much of each wavelength is affected individually (pseudo-colour image). B: Image captured after eight control loop iterations with no diffuser.

There were different reasons for the calibration errors, which we observed one due to physical imperfections, such as small curvatures on the display surface, which caused light refractive dispersion (smaller red dispersion, green and a bigger blue one). As seen in Fig 4A, in area 1 the RGB projections match the same pixels, which means that we can act in the desired place. However, RGB have a small offset between the different wavelengths of one pixel or two in area 2.

### Instability

The calibration errors of *^p^H_t_* could produce output instabilities. As stated, calibration errors were over 2 pixels, of which can occur in certain areas, which might move mapped pixels to dead zones (non-sensed dark zones). These calibration errors can cause an erroneous control by increasing the control action to the maximum, but without the mapped pixel detecting any change. Instead this control action can alter the measure in a pixel corresponding to a nearby texel, which could generate instabilities in the control (Fig 4B).

To avoid this problem, a diffuser was added to the display, which made the light diffuse obtaining bigger projected texels images (Fig 3C). Therefore, illumination with no dead zones was achieved and neighbouring pixels had a similar lighting level, which minimised the effects of calibration errors.

### Fixing the control action

**Fig 5:**
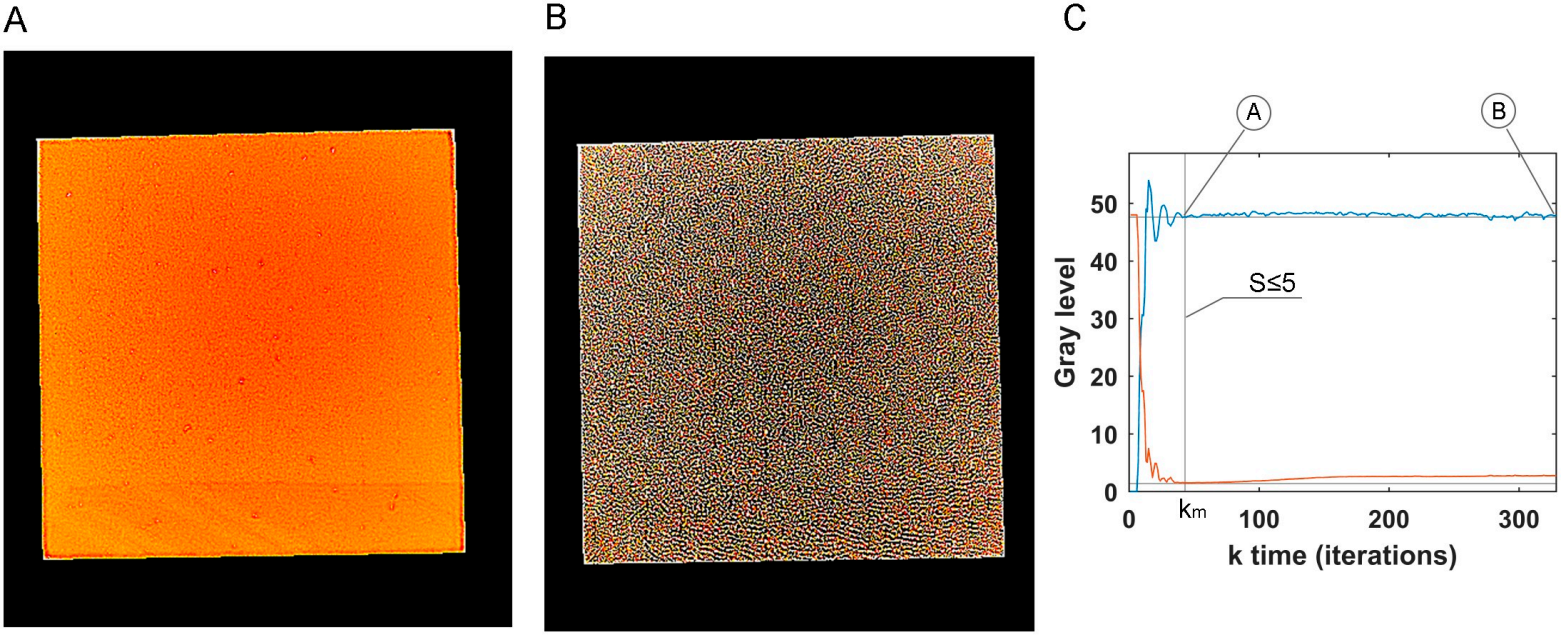
Stop control. (A) and (B) are the light patterns used as the control signal at different *k* instants when the error came close to zero, but (A) is the first one to reach it, and (B) is 283 iterations later. (C) Shows the output (blue) which is the grey level mean of image and control error to get reference.

The effectiveness of the fixing control action strategy was analysed with no Petri dish. Thus the control light pattern had no occlusion therefore it allowed each pixel on the image to reach the reference level (48). In these experiments, the control action on the display was applied continually over approximately 38*s* to compare the control error evolution of the pattern light applied at each time instant *k*. After a number of iterations *k*_*m*_ of the control loop, a stable control error was achieved henceforth. The period between the *k*_0_ and *k*_*m*_ control iteration is defined as transient period. After *k*_*m*_ iterations, the control lighting pattern tended to achieve a composition with maximum (white) and minimum (black) values (Fig 5B), although this provokes no change on the output image. This output image is maintained at the reference properly (a uniform grey image of 48 level) after *k*_*m*_. Hence, once the control error was estabilised, the control action was fixed (Fig 5A) henceforth. Fig 5C shows an experiment in which fixed control event is reached at *k*_*m*_ equals 45 iterations. Thus when (*S* ≤ 5) was detected, the control action was fixed. Fig 5A shows the fixed control lighting pattern at *k*_*m*_ equals 45 iterations. After performing different compensation processes, the time required to fix the control action to fall within the 6.16 ± 1.68*s* interval was estimated.

### Spatial variability

Some experiments were performed to analyse the quality of the images acquired with compensated at the 48 reference level and the uncontrolled standard white light. Several plates with NGM were tested by illuminating under the stated conditions. The images with background pixels at a grey level circa the 48 intensity level were expected when applying the light control to the system.

**Fig 6:**
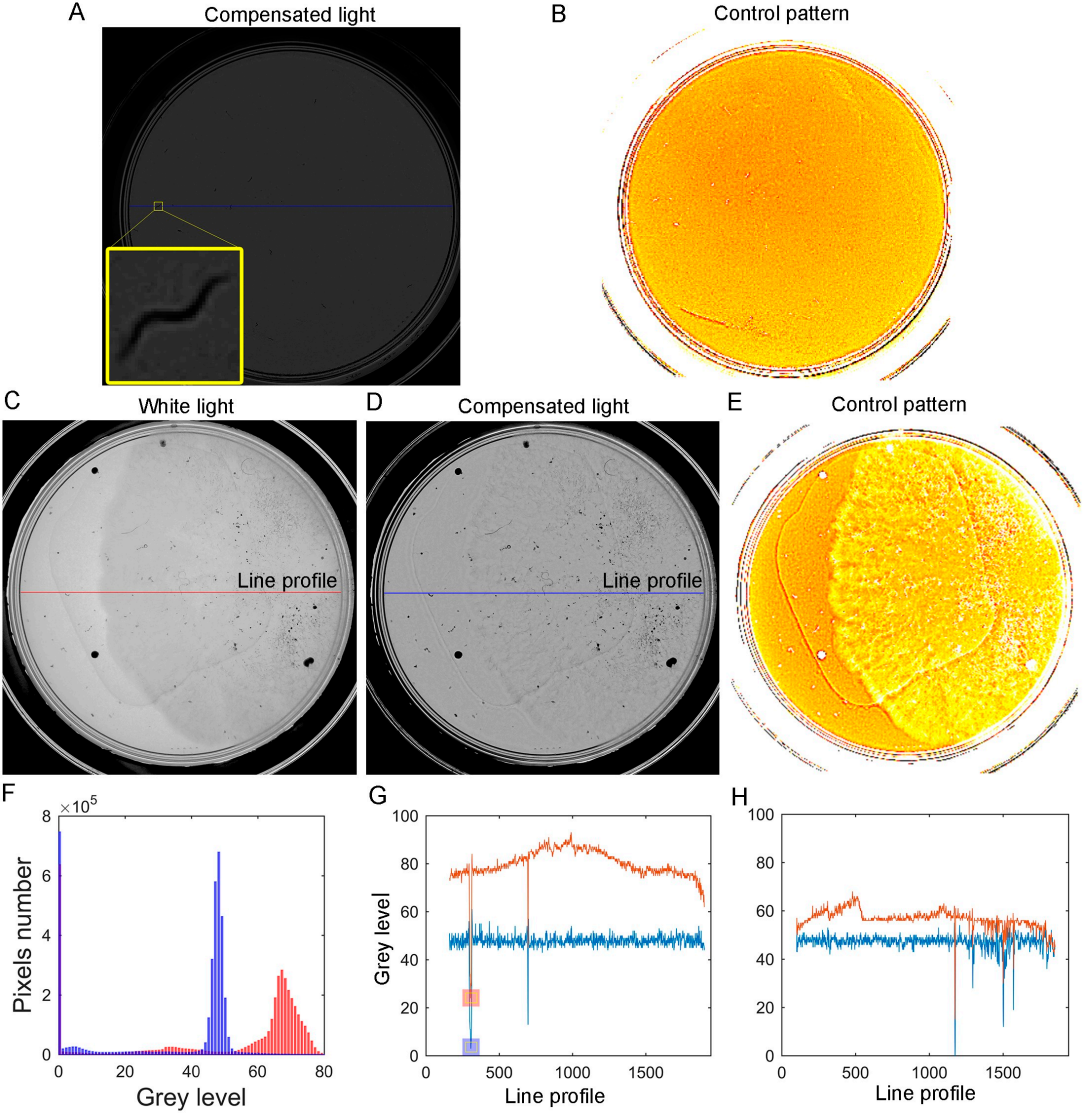
Spatial variability under white light. A: Captured image with compensated illumination. B: Control pattern applied to the captured image. C: Normalised image with white backlight. D: Normalised image with compensated light. E: Control pattern applied to the captured image (C). F: Comparison of the image histograms: the red one without applying any control and the blue one after applying the control. G: Comparison of profile lines, the red one without applying the control to (A) and the blue one after applying the control (A). The blue and red squares are the grey levels caused by a worm. H: Comparison of profile lines (C) and (D).

When applying the proposed illumination control strategy (Fig 6B and 6E), the captured images achieved nearly uniform illumination and improved image quality (Fig 6A and 6D). According to Fig 6F, when applying the illumination control, most of the background image pixels were around the 48 intensity level with worm pixels around the 0 level, as shown in the blue line profiles (Fig 6G and 6H). Fig 6C shows an image plate that underwent some kind of spatial intensity variations. When applying the control action (Fig 6E), these spatial variations were compensated (Fig 6D). The camera was configured at a low gain level and, therefore, images are dark. For this reason, images Fig 6C and 6D are seen to be normalised to simply improve their graph visualisation. The line profile on this contaminated plate (Fig 6H) could reach the reference in almost the whole area, but not all the contamination can be compensated. Some kind of opaque contamination was noted, which produced the low intensity level spots shown in Fig 6H.

Other experiments compared the first uncontrolled pattern light, used as the starting point in the active control loop, with the final compensated pattern light. The first pattern light was an orange pattern, which produced a constant grey image of the reference intensities (at level 48) with an ideal transparent medium.

**Fig 7:**
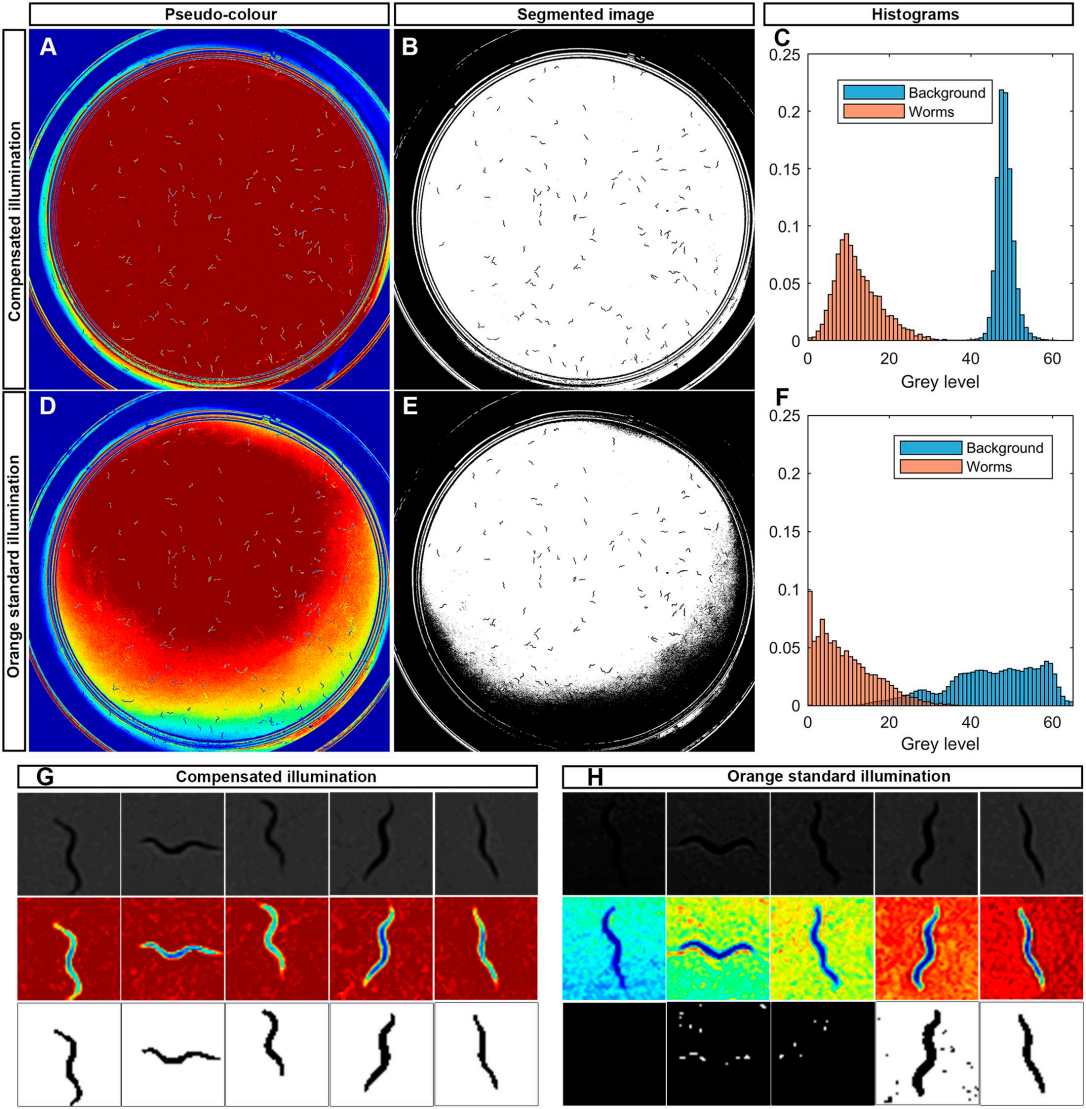
Spatial variability under orange light. A: Captured image pseudo-colour with compensated illumination. B: Segmented image of (A) by a threshold method. C: Histogram of grey level of background (blue) and worms (red) from (A). D: Captured image pseudo-colour with orange standard illumination. E: Segmented image of (D) by a threshold method. F: Histogram of grey level of background (blue) and worms (red) from (D). G: Worm image examples from (A) in pseudo-colour, grey scale and segmented. H: Worm image examples from (D) in grey scale, pseudo-colour and segmented.

It is well-known that the zones near the wall require greater light intensities than the plate centre. This effect is seen in Fig 7D. Therefore, if we used uncontrolled standard light, we would need to overlight the plate centre to properly illuminate the wall zones. However by using active light, we applied only the light required in each zone (Fig 7A). This generally reduces the light applied in the centre of the plate. It is stressed that active light would apply exactly the amount of light required to reach the reference. Images of worms can be more easily segmented using lower intensity light when applying the active control (Fig 7B) than uncontrolled light (Fig 7E).

In order to quantify image quality improvement, Fisher index was used [40], *F* (Eq (9)), which is a method that computes the importance of a feature (in our case the intensity levels) for segmentation in two classes (positive o negative). The respective score *F*(*j*) of feature *j* is given by:

$$\begin{array}{c} F\left(j\right)=|\frac{\mu_{j}^{+}-\mu_{j}^{-}}{{\left(\sigma_{j}^{+}\right)}^{2}+{\left(\sigma_{j}^{-}\right)}^{2}}| \end{array}$$ (9)

where $\mu_{j}^{+}\left(\mu_{j}^{-}\right)$ is the mean value for the *j*th feature in the positive (negative) class and $\sigma_{j}^{+}\left(\sigma_{j}^{-}\right)$ is the respective standard deviation.

We compared the mean and deviation between background intensities (histogram blue bars) and worms intensities (histogram red bars), for the uncontrolled standard light (Fig 7F) with the compensated light (Fig 7C). The compensated images show a higher Fisher index (0.8636 ± 0.1427) than the non-compensated images (0.2049 ± 0.0267). This is why the compensated images provide a better contrast in the zones near the wall and have, at the same time, a narrower variance than the non-compensated images. Fig 7G shows some examples of worms in several areas as the background is hold constantly by compensation, while the background of these same worms in the same areas has a wide variability for non-compensated illumination (Fig 7H). More examples can be found in S1 Fig.

### Temporal variability

**Fig 8:**
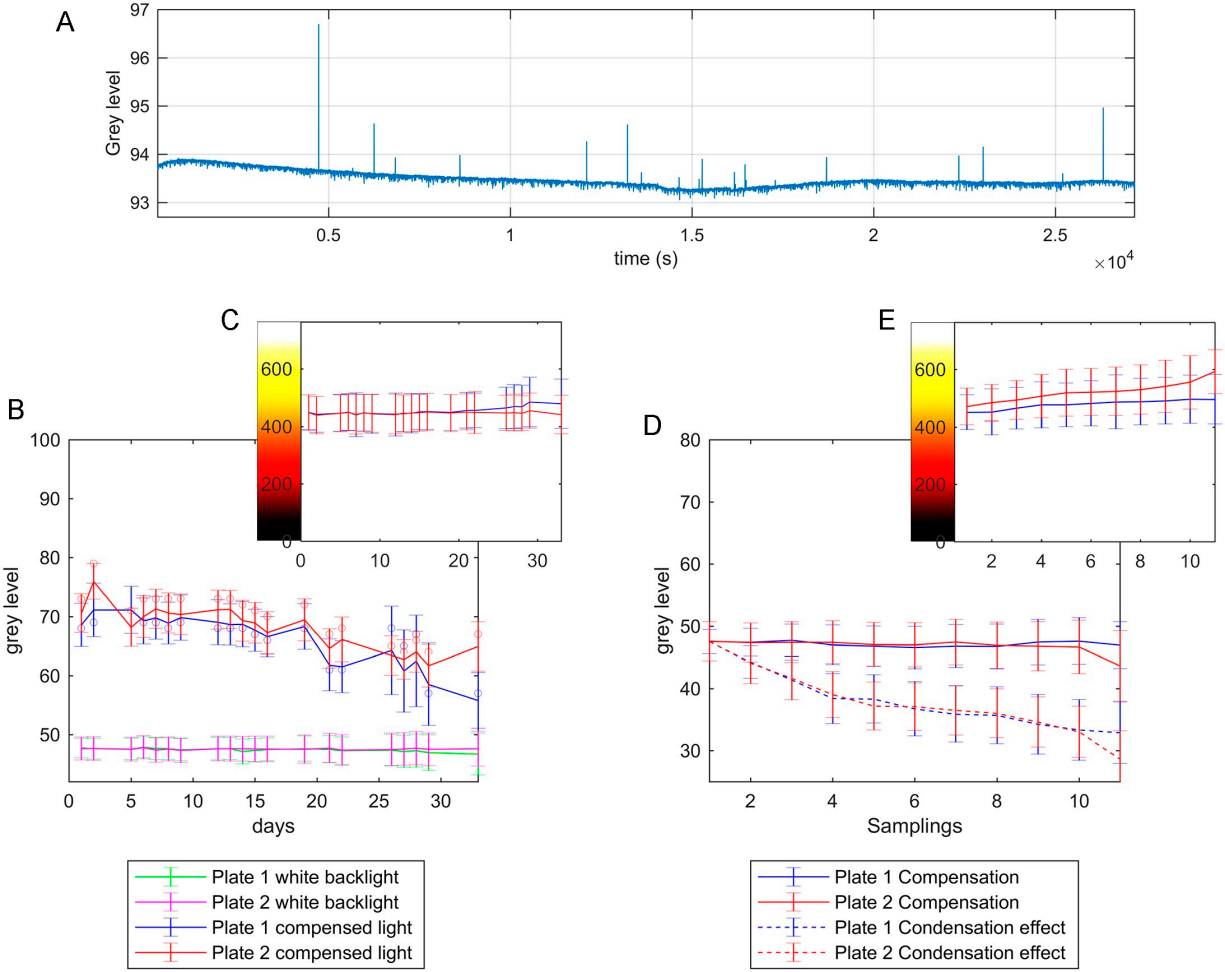
Temporal variability graphics. A: The average grey level of the image produced by a white backlight with time (about 30,000s). B: The mean grey levels and deviations of two plates for the white backlight and for the compensated light. C: The mean control signals and deviations applied for compensating images: (B) every day. D: The mean grey levels and deviations of two plates with condensation slowly increasing for the new backlight compensation every time that the mean image grey level error dropped below -3, and for the compensated light at the beginning (this lasted approximately 2h). E: The mean control signals and deviations applied for compensating the images in (D) during each new compensation event.

Temporal variations were studied in three experiments (Fig 8), which correspond to the three effects that may occur: illumination derivatives due to display fluctuations, media light characteristics which define that media have more or less opacity; condensation on lids, which causes occlusions.

An experiment to show that the source illumination derivatives were performed. In this case, light intensities were captured over a long time (7.5*h*). The camera and illumination source noise produced variation in intensities with time. Fig 8A shows there was a mean variation by noise with time at less than the ±0.25 grey level. However, there were also illumination derivatives of some peaks that were higher than noise (circa the Δ2 grey level) only for an instant. Display frequency was 60Hz and the camera was 8Hz. Thus the camera integration time was longer than the display refresh time and, therefore, the refresh action was not significant.

Another experiment was conducted to evaluate the effectiveness of the active illumination control strategy to compensate for medium variability. This experiment consisted of monitoring two Petri plates with solid medium (NGM) and *E. coli* strain OP50 for 33 days. Images were taken once a day (except for weekends) to observe the medium behaviour evolution with the non-compensated white backlight and the compensated backlight. All the media had different light transmittance characteristics, which presented temporal changes throughout the assays days. Fig 8B shows two media behaviours for 33 days, and an image grey level over time with a white backlight, from which the media’s increase opacity was deduced. When the control was applied, this variability was corrected by keeping the image mean value close to 48 every day, and its standard deviation at the ±2 grey level for the first 3 weeks. When no control was applied, the mean image grey level could vary from 70 grey levels to 55. Finally, in order to evaluate our active illumination control method over condensation, two Petri plates were monitored at an ambient room temperature of approximately 25°C for circa 2 hours. This time was necessary for condensation to occur. Plates were stored in an incubator at 20°C so that the rise in temperature in the room-warmed plates and condensation would be slowly evoked. At the zero time, and before any condensation was observed, a series of control actions was applied until the control error was estabilised. Then the last control action of this series was fixed and saved. Afterward, the error increased over time because condensation slowly appeared and made darker images. When this error was -3 intensity levels, we repeated the previous steps. A new series of control actions was applied until the error once again estabilised, and so on. Every time, and immediately before a new control action was fixed and saved, the control action of the first series was applied only to take one image. This series of images allowed to measure errors in relation to the first lighting compensation throughout the experiment. The control (Fig 8E) was also able to compensate for some soft condensation (Fig 8D). Condensation brought about a decrease of up to 18 grey levels in the mean, which could also be compensated.

## Discussion

First of all, it was important to analyse all the lighting factors that could affect image quality. Sometimes achieving a pure backlight illumination is difficult. We used a box to isolate the system from outside ambient lighting, but part of the light generated by our backlight bounced off on the box walls, with which a small ambient light component inherently appeared. Thus in order to reduce it, it was necessary to cover the interior with materials that were as little reflective as possible. It was proved that by covering the interior with black EVA rubber, ambient light reduced, which increased the contrast between worms and the background. This was verified by analysing the grey levels of worms, which went from 13 low levels to low ones of 0.

At the plate level, different reflections and refractions appeared on the standard Petri plate walls. Reflections could produce a mirror effect on the wall to show that reflected worms were near the walls. This was why our camera was configured at low sensitivity in an attempt to avoid these mirror effects. Regarding refractions, some light beams that passed through the Petri walls did not arrive at the camera, which produced dark circles near the walls (Fig 6). These areas were about 6% of the inspected area, which means that it was unlikely that a worm would be found in that areas because an *E. coli* lawn was strategically placed in the middle of the plate. Moreover, special segmentation software solutions can be applied for these areas.

Homography calibration was performed once at the setting up time with no Petri plate. So when the plate was inserted into the system, it provoked small refractions that increased the calibration errors. This error type could produce small intensity waves on the image when errors are too high. An alternative procedure would be to calibrate homography for each plate. However, the experiments showed that one calibration at the starting time with no plate was robust enough.

From the control point of view, there was a number of iterations *k* of the control loop, after which a stable lighting pattern was applied. This lighting pattern achieved a composition of maximum (white) and minimum (black) values (Fig 5B). However, to allow an effective wavelengths control strategy, the control action was fixed earlier when the control error was low (Fig 5A). We defined a detection event strategy to fix the control action, but other strategies can be used.

The strategic order (R, G, B) upon the control action was chosen after considering that the wavelengths near the ultraviolet are more detrimental for *C. elegans*’ survival. Therefore, blue light proved more detrimental than green light, and green light was more detrimental than red light [32]. Given this strategy, most of the plate was illuminated with light that was close to orange (255, 190, 0), as seen in Fig 8C and 8D. The maximum control action, white light (255, 255, 255), was applied automatically, but only to opaque zones (Petri walls and strong contamination) and worms. As it was not possible to detect worms in opaque zones, applying blue light to these zones is a very interesting strategy because worms tend to avoid blue-illuminated zones [33]. In addition, applying blue light to worms stimulates their movement, which could improve lifespan results. In our case, the maximum intensity level of the blue light was applied to worms because the monitoring process lasted only few seconds. Depending on the application, other control strategies can also be implemented by applying light as desired.

To avoid control instabilities due to calibration errors, stable PIDs regulators were introduced and a diffuser was added to the display. Another solution without a diffuser would be to measure output (*y*_*k*_) as the average of 30 pixels, which was related to the same texel. However, if we used a diffuser, we would only have to take the central pixel, and in such a way that the computational load of all these means calculations would be avoided. When using a diffuser, a new problem could arise due to the texel lighting affecting not only the pixels associated with it in calibration, but also those associated with neighbouring texels. Hence it became a coupled system by conferring interactions in the control. In order to control these interactions (Fig 3C), a controller with a small *k*_*p*_ was designed. If a faster settling time is required, these interactions can be modelled to apply advanced controllers.

The experimental results show that this method is able to compensate for automatically different illumination changes. This changes involve media transparency on all the assay days, whose mean image grey level can vary from 70 to 55 (Fig 8B); changes in ambient conditions, such as smooth condensation on lids, whose mean image grey level can vary by 18 grey levels (Fig 8D); light derivatives of the illumination source during its lifetime. Therefore, the proposed control technique simplifies the worms segmentation problem by reaching near uniform illumination throughout the image. This might both increase the quality in all areas (by reducing information loss) and obtain constant illumination in the whole area (by allowing fixed threshold image segmentation).

To a greater or lesser extent, uneven lighting is quite a common problem when monitoring *C. elegans* and other organisms, such as *Saccharomyces cerevisiae*, *zebrafish larvae* and *Drosophila larvae* cultured in Petri plates systems. The new proposed method compensates for some spatial and temporal changes, and makes segmentation easier and more efficient. This method can not only be used in lifespan or healthspan assays, but it should also serve a broad range of applications in optogenetics.

Future research could improve system accuracy by calculating three different homographies, one for each wavelength (R, G, B), which would reduce calibration errors. In addition, this system could be implemented with other lighting matrices with other wavelengths and intensities. Finally, other regulators like predictive control could also be implemented.

## Supporting information

S1 Video Example video of method. Here is an example video of method performance.
(MP4)

S1 Fig Worm images. More worm images captured, which are pseudo-colour where blue is the darkest grey level value and red is the 48 grey level.
(TIF)

S1 Appendix Code. In this section is the code we developed. It is C++ program for raspbian operating system running on a Raspberry Pi. https://github.com/JCPuchalt/c-elegans_smartLight.
(RAR)

S2 Appendix Hardware assembly. In this section is detailed (1) software configuration; (2) mechanical assembly; (3) display, camera and processor are wired etc.
(PDF)

## References

[1] Brenner S. THE GENETICS OF CAENORHABDITIS ELEGANS. Genetics. 1974;77(1):71–94. 4366476 10.1093/genetics/77.1.71 PMC1213120

[2] Tissenbaum HA. Using C. elegans for aging research. Invertebrate Reproduction & Development. 2015;59(sup1):59–63. 10.1080/07924259.2014.940470 26136622 PMC4464094

[3] Kenyon CJ. The genetics of ageing. Nature. 2010;464:504 10.1038/nature08980 20336132

[4] Guarente L, Kenyon C. Genetic pathways that regulate ageing in model organisms. Nature. 2000;408:255 10.1038/35041700 11089983

[5] Walker DW, McColl G, Jenkins NL, Harris J, Lithgow GJ. Evolution of lifespan in C. elegans. Nature. 2000;405(6784):296–297. 10.1038/35012693 10830948

[6] Amrit FRG, Ratnappan R, Keith SA, Ghazi A. The C. elegans lifespan assay toolkit. Methods. 2014;68(3):465–475. 10.1016/j.ymeth.2014.04.002 24727064

[7] Klass MR. Aging in the nematode Caenorhabditis elegans: Major biological and environmental factors influencing life span. Mechanisms of Ageing and Development. 1977;6:413–429. 10.1016/0047-6374(77)90043-4 926867

[8] Aitlhadj L, Stürzenbaum SR. The use of FUdR can cause prolonged longevity in mutant nematodes. Mechanisms of Ageing and Development. 2010;131(5):364–365. 10.1016/j.mad.2010.03.002 20236608

[9] Hosono R. Age dependent changes in the behavior of Caenorhabditis elegans on attraction to Escherichia coli. Experimental Gerontology. 1978;13(1):31–36. 10.1016/0531-5565(78)90027-X 346360

[10] Hosono R. Sterilization and growth inhibition of Caenorhabditis elegans by 5-fluorodeoxyuridine. Experimental Gerontology. 1978;13(5):369–373. 10.1016/0531-5565(78)90047-5 153845

[11] Onken B, Driscoll M. Metformin Induces a Dietary Restriction–Like State and the Oxidative Stress Response to Extend C. elegans Healthspan via AMPK, LKB1, and SKN-1. PLOS ONE. 2010;5(1):1–13. 10.1371/journal.pone.0008758 PMC2807458 20090912

[12] Keith SA, Amrit FRG, Ratnappan R, Ghazi A. The C. elegans healthspan and stress-resistance assay toolkit. Methods. 2014;68(3):476–486. 10.1016/j.ymeth.2014.04.003 24727065

[13] Hahm JH, Kim S, DiLoreto R, Shi C, Lee SJV, Murphy CT, et al C. elegans maximum velocity correlates with healthspan and is maintained in worms with an insulin receptor mutation. NATURE COMMUNICATIONS. 2015;6 10.1038/ncomms9919 26586186 PMC4656132

[14] Hertweck M, Baumeister R. Automated assays to study longevity in C. elegans. In: Mechanisms of Ageing and Development. vol. 126; 2005 p. 139–145. 10.1016/j.mad.2004.09.010 15610772

[15] Swierczek NA, Giles AC, Rankin CH, Kerr RA. High-throughput behavioral analysis in C. elegans. Nature Methods. 2011;8(7):592–U112. 10.1038/nmeth.1625 21642964 PMC3128206

[16] Stroustrup N, Ulmschneider BE, Nash ZM, López-Moyado IF, Apfeld J, Fontana W. The Caenorhabditis elegans Lifespan Machine. Nature methods. 2013;10(7):665–70. 10.1038/nmeth.2475 23666410 PMC3865717

[17] Puckering T, Thompson J, Sathyamurthy S, Sukumar S, Shapira T, Ebert P. Automated Wormscan. F1000Research. 2017;6:192 10.12688/f1000research.10767.1 28413617 PMC5365223

[18] Fontaine E, Burdick J, Barr A. Automated Tracking of Multiple C. Elegans. In: 2006 International Conference of the IEEE Engineering in Medicine and Biology Society; 2006. p. 3716–3719. 10.1109/IEMBS.2006.260657 17945791

[19] Jung SK, Aleman-Meza B, Riepe C, Zhong W. QuantWorm: A comprehensive software package for Caenorhabditis elegans phenotypic assays. PLoS ONE. 2014;9(1). 10.1371/journal.pone.0084830 PMC3885606 24416295

[20] Roussel N, Morton CA, Finger FP, Roysam B. A Computational Model for C. elegans Locomotory Behavior: Application to Multiworm Tracking. IEEE Transactions on Biomedical Engineering. 2007;54(10):1786–1797. 10.1109/TBME.2007.894981 17926677

[21] Mathew MD, Mathew ND, Ebert PR. WormScan: A Technique for High-Throughput Phenotypic Analysis of Caenorhabditis elegans. PLOS ONE. 2012;7(3). 10.1371/journal.pone.0033483 PMC3311640 22457766

[22] Otsu N. A Threshold Selection Method from Gray-Level Histograms. IEEE Transactions on Systems, Man, and Cybernetics. 1979;9(1):62–66. 10.1109/TSMC.1979.4310076

[23] Wählby C, Kamentsky L, Liu ZH, Riklin-Raviv T, Conery AL, O’Rourke EJ, et al An image analysis toolbox for high-throughput C. elegans assays. Nature methods. 2012;9(7):714–6. 10.1038/nmeth.1984 22522656 PMC3433711

[24] Chen W, Liao B, Li W, Dong X, Flavel M, Jois M, et al Segmenting Microscopy Images of Multi-Well Plates Based on Image Contrast. Microscopy and Microanalysis. 2017;23(5):932–937. 10.1017/S1431927617012375 28712372

[25] Kainmueller D, Jug F, Rother C, Myers G. Active Graph Matching for Automatic Joint Segmentation and Annotation of C. elegans BT—Medical Image Computing and Computer-Assisted Intervention—MICCAI 2014. Cham: Springer International Publishing; 2014 p. 81–88. 10.1007/978-3-319-10404-1_11 25333104

[26] Raviv TR, Ljosa V, Conery AL, Ausubel FM, Carpenter AE, Golland P, et al Morphology-Guided Graph Search for Untangling Objects: C. elegans Analysis BT—Medical Image Computing and Computer-Assisted Intervention—MICCAI 2010. Berlin, Heidelberg: Springer Berlin Heidelberg; 2010 p. 634–641. 10.1007/978-3-642-15711-0_79 PMC3050593 20879454

[27] Tsechpenakis G, Bianchi L, Metaxas DN, Driscoll M. A novel computational approach for simultaneous tracking and feature extraction of C. elegans populations in fluid environments. IEEE Transactions on Biomedical Engineering. 2008;55(5):1539–1549. 10.1109/TBME.2008.918582 18440900

[28] Cronin CJ, Mendel JE, Mukhtar S, Kim YM, Stirbl RC, Bruck J, et al An automated system for measuring parameters of nematode sinusoidal movement. BMC GENETICS. 2005;6 10.1186/1471-2156-6-5 15698479 PMC549551

[29] Restif C, Ibáñez-Ventoso C, Vora MM, Guo S, Metaxas D, Driscoll M. CeleST: Computer Vision Software for Quantitative Analysis of C. elegans Swim Behavior Reveals Novel Features of Locomotion. PLoS Computational Biology. 2014;10(7). 10.1371/journal.pcbi.1003702 25033081 PMC4102393

[30] Geng W, Cosman P, Baek JH, Berry CC, Schafer WR. Quantitative Classification and Natural Clustering of Caenorhabditis elegans Behavioral Phenotypes. Genetics. 2003;165(3):1117 LP—1126. 14668369 10.1093/genetics/165.3.1117 PMC1462821

[31] Geng W, Cosman P, Berry CC, Feng Z, Schafer WR. Automatic tracking, feature extraction and classification of C. elegans phenotypes. IEEE Transactions on Biomedical Engineering. 2004;51(10):1811–1820. 10.1109/TBME.2004.831532 15490828

[32] De Magalhaes Filho CD, Henriquez B, Seah NE, Evans RM, Lapierre LR, Dillin A. Visible light reduces C. elegans longevity. Nature Communications. 2018;9(1). 10.1038/s41467-018-02934-5 29500338 PMC5834526

[33] Edwards SL, Charlie NK, Milfort MC, Brown BS, Gravlin CN, Knecht JE, et al A novel molecular solution for ultraviolet light detection in Caenorhabditis elegans. PLOS BIOLOGY. 2008;6(8):1715–1729. 10.1371/journal.pbio.0060198 PMC2494560 18687026

[34] Lee KH, Aschner M. A Simple Light Stimulation of Caenorhabditis elegans In: Current Protocols in Toxicology. John Wiley & Sons, Inc; 2001. Available from: 10.1002/0471140856.tx1121s67. PMC4747329 26828328

[35] Churgin MA, Jung SK, Yu CC, Chen X, Raizen DM, Fang-Yen C. Longitudinal imaging of Caenorhabditis elegans in a microfabricated device reveals variation in behavioral decline during aging. eLife. 2017;6 10.7554/eLife.26652 PMC5484621 28537553

[36] Ricolfe-Viala C, Sánchez-Salmerón AJ. Using the camera pin-hole model restrictions to calibrate the lens distortion model. Optics and Laser Technology. 2011;43(6):996–1005. 10.1016/j.optlastec.2011.01.006

[37] Percoco G, Lavecchia F, Salmerón AJS. Preliminary study on the 3D digitization of millimeter scale products by means of photogrammetry. In: Procedia CIRP. vol. 33; 2015 p. 257–262. 10.1016/j.procir.2015.06.046

[38] Juchem J, Lefebvre S, Mac Thi T, Ionescu CM. An analysis of dynamic lighting control in landscape offices. In: Preprints of the 3rd IFAC Conference on Advances in Proportional-Integral-Derivative Control (PID); 2018. p. 232–237.

[39] Keyser RD, Ionescu C. Modelling and simulation of a lighting control system. Simulation Modelling Practice and Theory. 2010;18(2):165–176. 10.1016/j.simpat.2009.10.003

[40] Maldonado S, Weber R. A wrapper method for feature selection using Support Vector Machines. Information Sciences. 2009;179(13):2208–2217. 10.1016/j.ins.2009.02.014

