# Supplementary figures and images for: Active backlight for automating visual monitoring: An analysis of a lighting control technique for Caenorhabditis elegans cultured on standard Petri plates
Source: PLoS One. 2019 Apr 16;14(4):e0215548. doi: 10.1371/journal.pone.0215548 (PMC6467411; doi:10.1371/journal.pone.0215548)

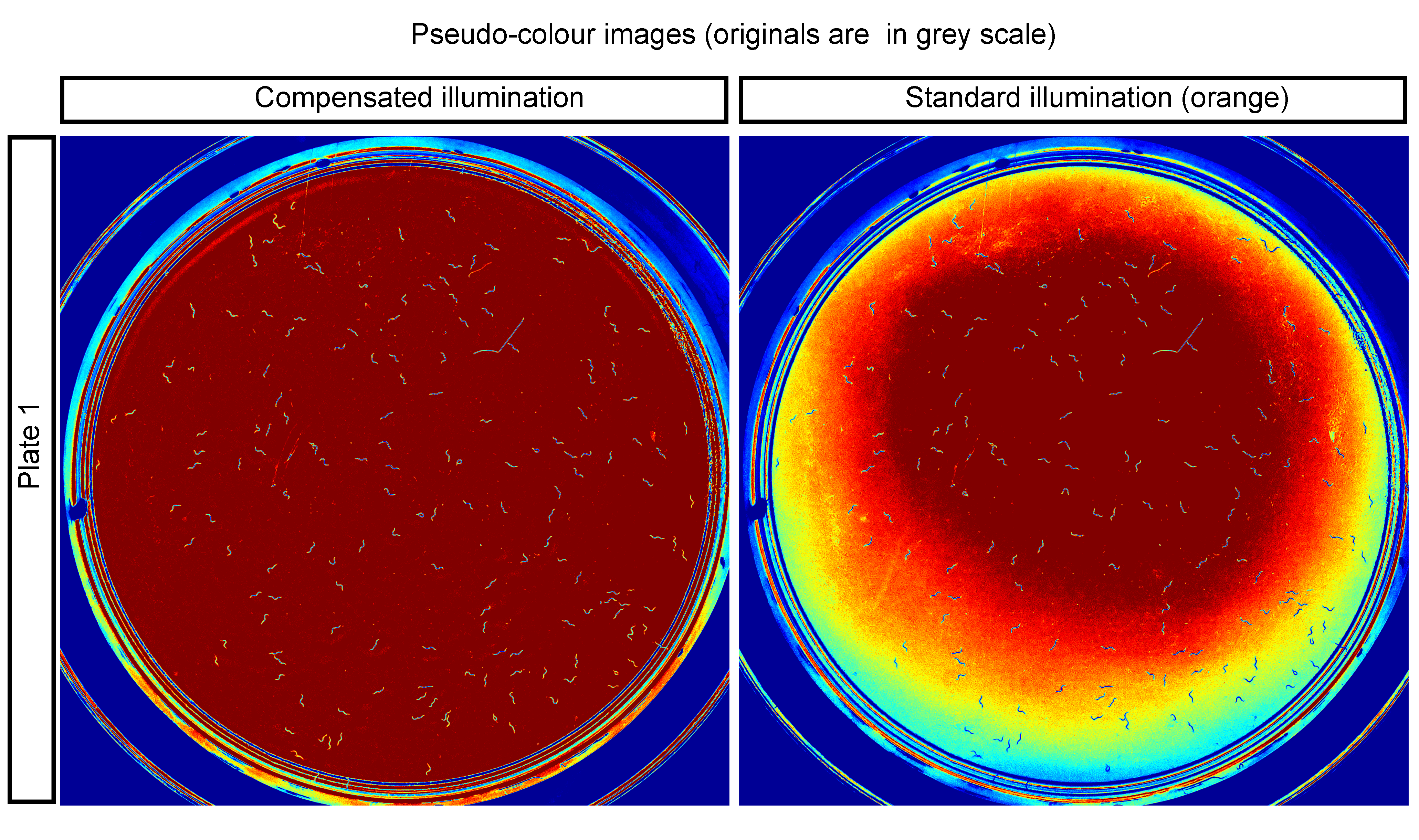

Supplement: S1 Fig — More worm images captured, which are pseudo-colour where blue is the darkest grey level value and red is the 48 grey level. (TIF) [file pone.0215548.s002.tif]
