# Supplementary material for: Active backlight for automating visual monitoring: An analysis of a lighting control technique for Caenorhabditis elegans cultured on standard Petri plates
Source: PLoS One. 2019 Apr 16;14(4):e0215548. doi: 10.1371/journal.pone.0215548 (PMC6467411; doi:10.1371/journal.pone.0215548)
Supplement: S2 Appendix — In this section is detailed (1) software configuration; (2) mechanical assembly; (3) display, camera and processor are wired etc. (PDF) [file pone.0215548.s004.pdf]

Electronics

- Raspberry Pi 3
- Micro SD card (more than 16GB)
- Raspberry Pi power supply at least 2A
- Pi NoIR Camera V1
- 7" Raspberry Pi Display

Assembly hardware

A method is presented, therefore the hardware mays have any configuration. As a proof of concept is suggested following assembly, nevertheless may be completely different.

First shutdown system and disconnect power supply.

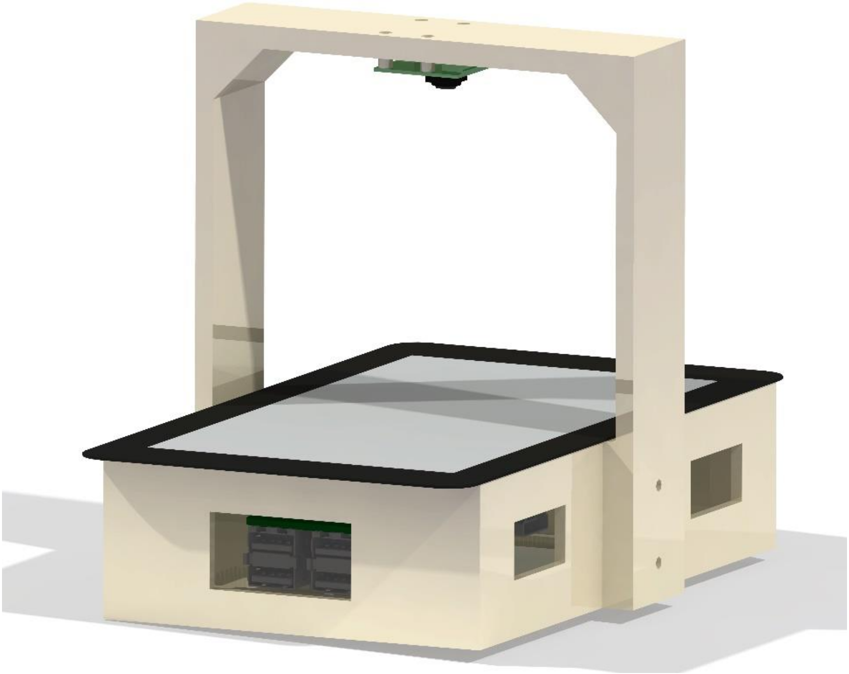

Assembly bridge (red part) and display box (blue part) by screwing in holes.

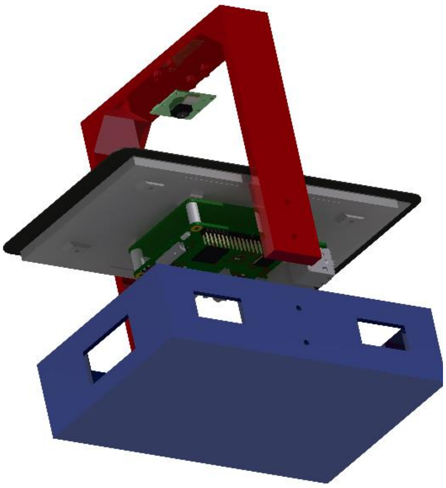

- Bridge is the red piece, it is used to place the camera, which is screwed to the bridge.
- The blue piece encloses the electronics and holds the display.
- The red and blue part of the drawing must be screwed in, thus the coordinate axes of the display and the camera maintain the reference between them.

Bridge https://github.com/JCPuchalt/c-elegans_smartLight/blob/master/3DModel/Bridge.stl

DisplayBox https://github.com/JCPuchalt/c-elegans_smartLight/blob/master/3DModel/DisplayBox.stl

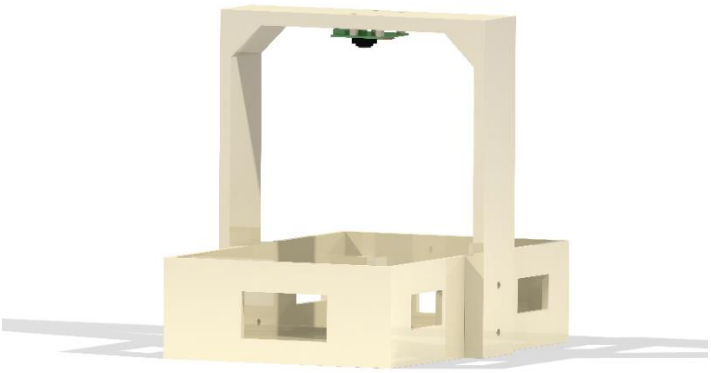

Wiring

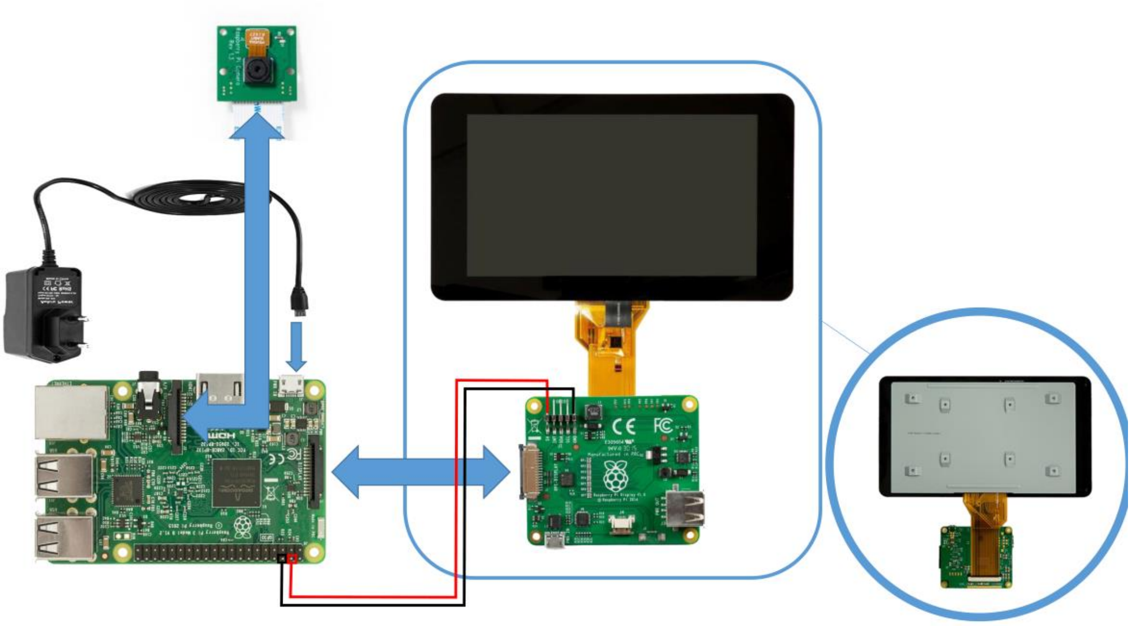

1. Connect the wide ribbon cable to the underside of the display board (Fig. 1A).
2. Connect the small ribbon cable to the connector on the top of the board (Fig. 1B).

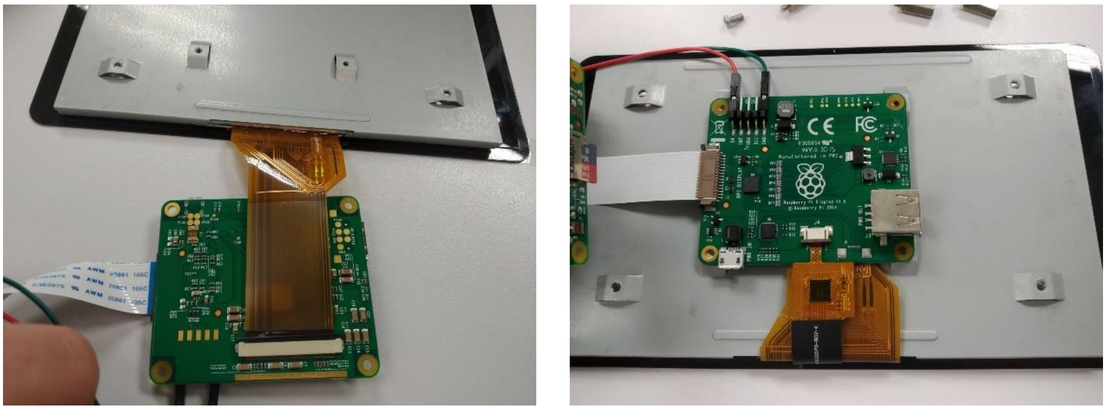

Fig 1. (A) Left. (B) Right.

3. Screw the driver to the display (Fig 2)

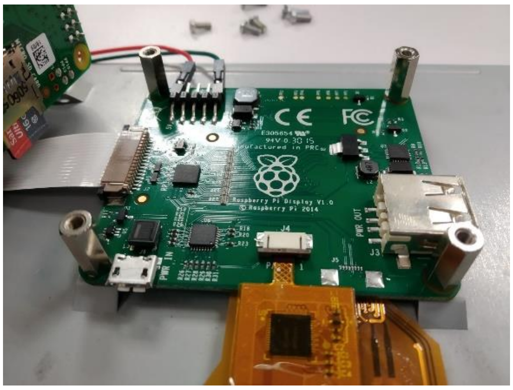

Fig 2.

Connect driver to Raspberry Pi

4. Connect the two jumper wires to the 5V and GND pins on the display driver board (Fig 2).
5. Take the white ribbon cable and connect it to the connector on the board. The blue mark on one end towards the back of the screen (Fig 2).
6. Place the SD card in Raspberry Pi (Fig. 3A) and screw Raspberry Pi to the driver (Fig. 3B) .

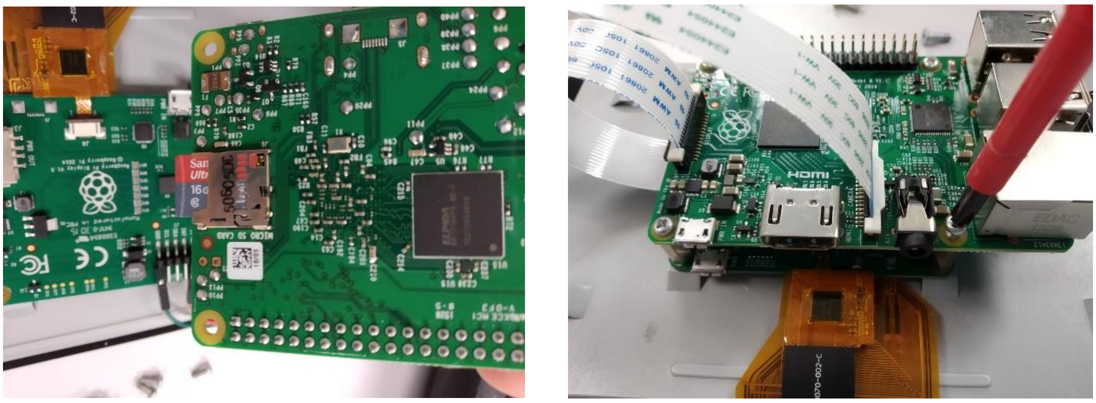

Fig 3. (A) Left. (B) Right.

7. Connect the white ribbon cable to the Raspberry display connector (Fig. 4A).
8. Connect the two jumper wires to the 5V and GND pins on the GPIO (Fig. 4B).

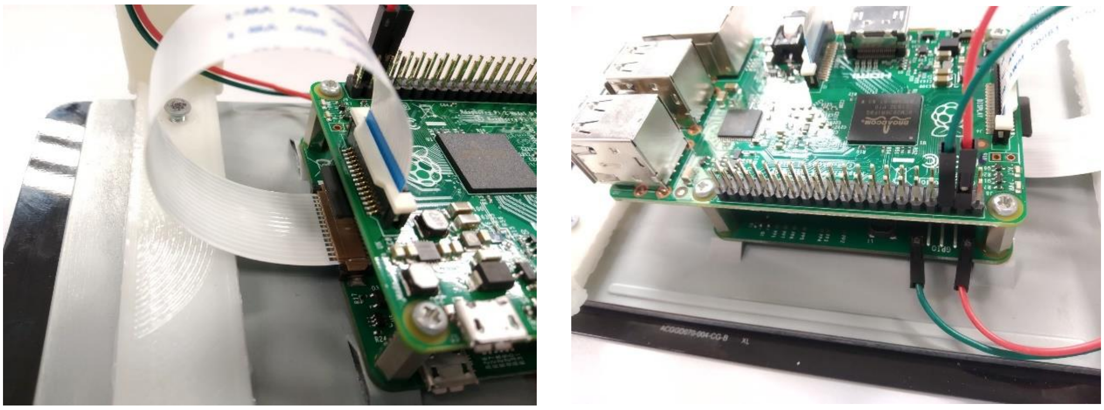

Fig 4. (A) Left. (B) Right.

9. Connect Ethernet cable (Fig. 5).

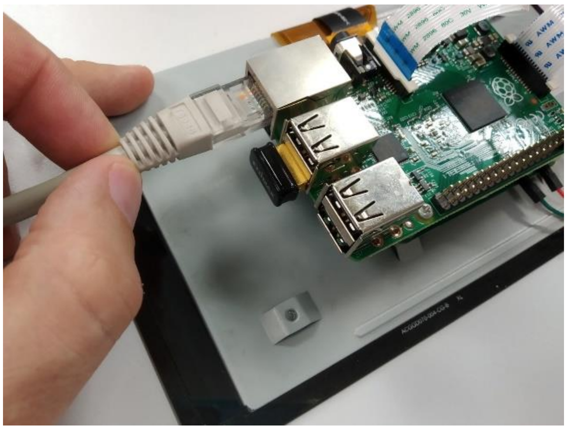

Fig. 5

10. Connect white ribbon cable for the camera to Raspberry camera connector (Fig. 6A). Cable length must have more than 20cm.
11. Connect the power supply that is able to supply at least 2 amps (Fig. 6B).

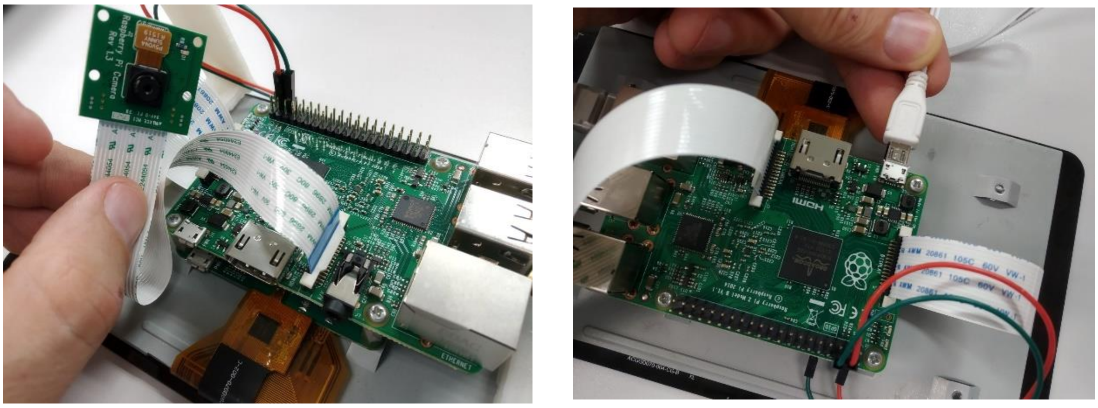

Fig 6. (A) Left. (B) Right.

12. Complete assembly (Fig. 7A).
13. Turn the display over (Fig. 7B).

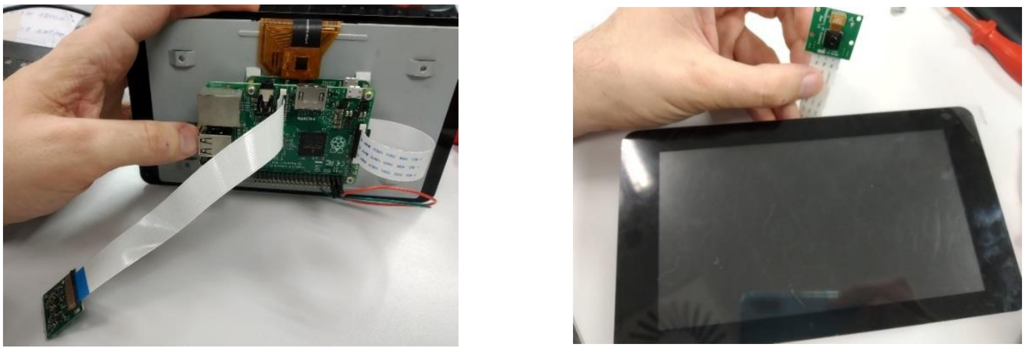

Fig 7. (A) Left. (B) Right.

14. Wait for the device to turn on.

Install Operating System Raspbian on Raspberry Pi

- Download from https://www.raspberrypi.org/downloads/noobs/
- Unzip files
- Copy the unzip files to the formatted Micro SD card
- Place the Micro SD card into Micro SD card slot on the Raspberry Pi
- Connect any display to Raspberry Pi
  - Through HDMI for any display
  - Through display connector for Raspberry display (see details below)
- Connect keyboard to USB port
- Follow the installation steps

Once installed, write down the IP address shown on screen as may see on Fig. 8B (in this case 192.168.1.184).

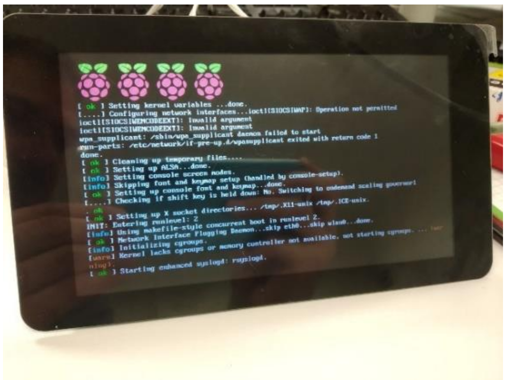

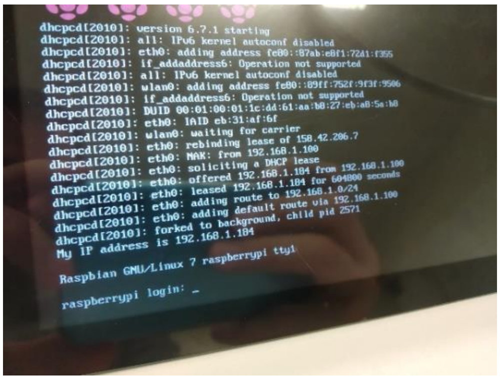

Fig. 8. (A) Left. (B) Right.

And login:

USER: pi
PASSWORD: raspberry

Set up system

There is a script to easy installation and configuration (follow steps) or details for configuration and installation in next steps.

Raspberry Pi configuration

```
sudo raspi-config
```

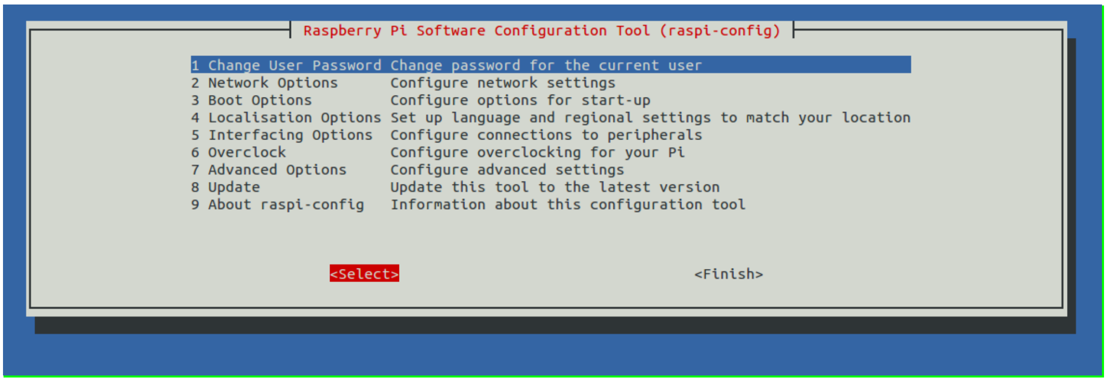

- Select: Interfacing options -> Camera (enable)
- Select: Interfacing options -> SSH (enable)
- Set memory split for GPU to 250MB (Advanced options -> Memory split)

Update the system before adding new hardware or installing new software.

- sudo apt-get update
- sudo apt-get upgrade

Requirements

- Install cmake
- Install ncurses
- OpenCV and opencv_contrib
- Install userland in [/home/pi/userland] (https://github.com/raspberrypi/userland)

Installation c-elegans_smartLight program

Copy code in a folder (i.e. [/home/pi/ c-elegans_smartLight])

```
cd c-elegans_smartLight
cmake .
make
```

Run program

1. Connect from PC through SSH. **USER:** pi. **IP:** 192.168.1.184. **PASSWORD:** raspberry.
    a. Linux -> On terminal: **ssh USER@IP_ADDRESS** (ssh pi@192.168.1.184)
    b. Windows -> ssh program (i.e. PuTTY)
2. Run program on terminal.

```
cd c-elegans_smartLight
sudo ./celegans
```

Camera (must be Pi NoIR Camera V1)

Focus the camera by turning the lens (A), it may be used command `raspistill -t 0` (when being finished press ctrl + c). Be careful when turning the lens (A), do not scrape it, and hold it steady B, do not twist. The camera should be coplanar to the display focusing at the centre of it at a distance of about 8cm. Make sure that the calibration pattern (Fig. 10) is completely captured with the camera. To move it you can modify it with the file "./calibracio/p1.xml".

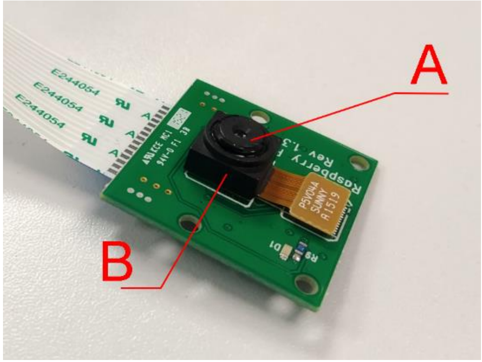

Fig. 9

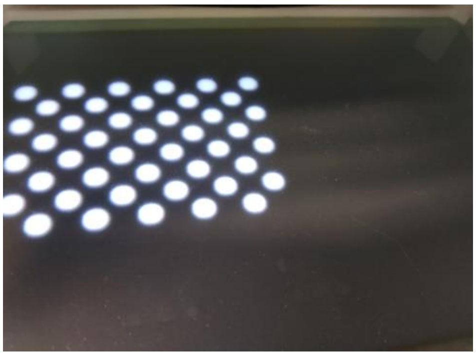

Fig. 10
